# Physiological and pathophysiological functions of cell cycle proteins in post-mitotic neurons: *implications for Alzheimer’s disease*

**DOI:** 10.1007/s00401-015-1382-7

**Published:** 2015-01-25

**Authors:** Lucie A. G. van Leeuwen, Jeroen J. M. Hoozemans

**Affiliations:** Department of Pathology, VU University Medical Center, Neuroscience Campus Amsterdam, PO Box 7057, 1007 MB Amsterdam, The Netherlands

**Keywords:** Alzheimer’s disease, Cell cycle, DNA repair, Neurodegeneration, Neuroplasticity

## Abstract

Alzheimer’s disease (AD) is the most prevalent neurodegenerative disorder for which no effective treatment is available. Increased insight into the disease mechanism in early stages of pathology is required for the development of a successful therapy. Over the years, numerous studies have shown that cell cycle proteins are expressed in neurons of AD patients. Traditionally, neurons are considered to be post-mitotic, which means that they permanently retract from the cell cycle. The expression of cell cycle proteins in adult neurons of AD patients has therefore been suggested to promote or even instigate pathomechanisms underlying AD. Interestingly, expression of cell cycle proteins is detected in post-mitotic neurons of healthy controls as well, albeit to a lesser extent than in AD patients. This indicates that cell cycle proteins may serve important physiological functions in differentiated neurons. Here, we provide an overview of studies that support a role of cell cycle proteins in DNA repair and neuroplasticity in post-mitotic neurons. Aberrant control of these processes could, in turn, contribute to cell cycle-mediated neurodegeneration. The balance between regenerative and degenerative effects of cell cycle proteins in post-mitotic neurons might change throughout the different stages of AD. In the early stages of AD pathology, cell cycle protein expression may primarily occur to aid in the repair of sublethal double-strand breaks in DNA. With the accumulation of pathology, cell cycle-mediated neuroplasticity and neurodegeneration may become more predominant. Understanding the physiological and pathophysiological role of cell cycle proteins in AD could give us more insight into the neurodegenerative process in AD.

## Introduction

Alzheimer’s disease (AD), the most prevalent neurodegenerative disorder, is responsible for the majority of late-onset dementia cases [8]. The disease follows a progressive and fatal disease course. Pathological hallmarks of AD include severe neurodegeneration, senile plaques consisting of extracellular deposits of amyloid-β (Aβ) protein, and neurofibrillary tangles (NFTs) composed of intracellular aggregates of hyperphosphorylated microtubule associated tau protein. Aβ protein is a proteolytic fragment of the β-amyloid precursor protein (APP). Autosomal dominantly inherited mutations in the genes that encode for APP, presenilin 1 (PSEN1) and presenilin 2 (PSEN2) contribute to enhanced deposition of Aβ and are causally associated with an early onset of AD. Mutations identified in APP are missense mutations lying within or close to the domain encoding the Aβ peptide. Mutations in PSEN1 and PSEN2 directly affect the proteolysis of APP leading to increased levels of Aβ [101]. Autosomal dominant inherited mutations leading to AD are relatively uncommon. More common is the occurrence of a variant of the gene encoding for apolipoprotein E, APOE-ε4, which has been shown to be a risk factor for AD and occurs in both early and late-onset cases [93]. APOE-ε4 also contributes to the accumulation of Aβ, as shown in studies with transgenic mice [76, 92]. More support for a central role of Aβ in the pathogenesis of AD comes from the observation that patients with Down’s syndrome show increased risk of dementia and cerebral Aβ deposits at early age. In Down’s syndrome the abundant cerebral deposition of Aβ is attributed to excess APP synthesis due to the extra copy of chromosome 21 where the APP gene is located. The pathogenic effects of mutations associated with early-onset AD have strongly contributed to the hypothesis that AD is an amyloid driven process. The widely accepted, but not undisputed, amyloid cascade hypothesis, proposes that Aβ drives neuritic tau pathology, being an important secondary phenomenon that is closely correlated with the syndrome of dementia [34, 85]. Despite exhaustive knowledge about the various neuropathological correlates of AD, as of yet no general consensus has been reached regarding the mechanism underlying neuronal dysfunction and neuronal loss in AD, especially in the prodromal phase of the disease. This has made the development of disease models and therapies extremely difficult.

Presently, post-mortem studies have reported increased expression of cell cycle proteins in post-mitotic neurons of AD patients. The cell cycle has been linked to all abovementioned AD hallmarks, and it has therefore been put forward that neuronal cell cycle re-entry may promote AD pathology and that a failure of completing the cell cycle results in neurodegeneration, a phenomenon referred to as ‘abortosis’ [77]. However, as will be discussed below, cell cycle proteins are also expressed in neurons without apparent pathological changes and neurons of healthy non-demented individuals. Here, we will review physiological and pathophysiological roles of cell cycle proteins in post-mitotic neurons. Recognizing the events that could drive a post-mitotic neuron to re-express cell cycle proteins will help us interpret the significance of these proteins in AD.

## Cell cycle proteins in AD post-mortem brain tissue

**Fig. 1:**
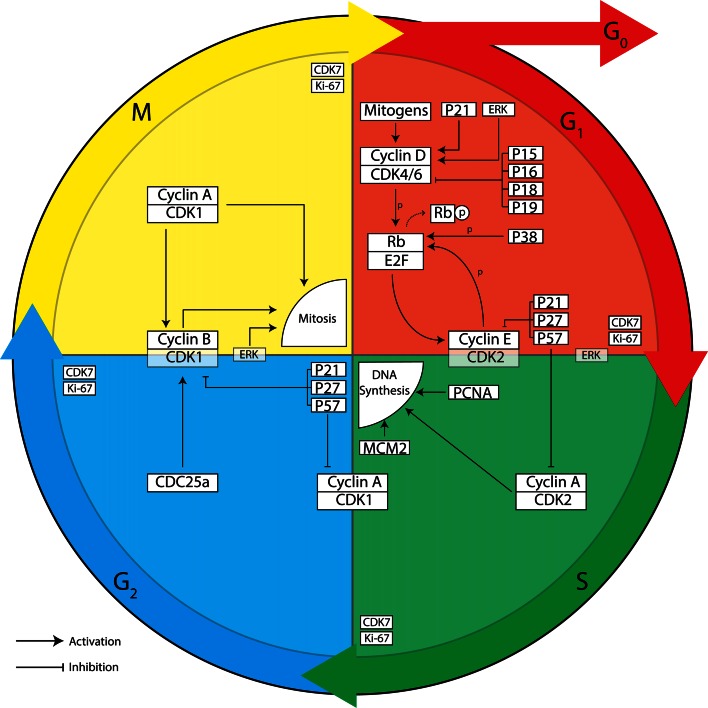
Overview of proteins involved in regulation of the cell cycle. The cell division cycle of eukaryotic cells can be divided into four phases. During *S* phase, DNA synthesis takes place and during *M* phase mitosis and cytokinesis occurs. *G*_*1*_ and *G*_*2*_ are gap phases, which separate *S* phase and *M* phase. Cells can enter a permanent resting state, referred to as *G*_*0*_ phase. Neurons continuously reside in *G*_*0*_ phase. Progression through the cell cycle is tightly governed by the cell cycle control system, consisting of cyclin-dependent kinases (CDK), cyclins and CDK inhibitors (CDKIs). CDKs need to bind to cyclins to become catalytically active. CDK-cyclin complexes important for phase transition are depicted on the *border of two phases*. Important inhibitors of cyclin D-CDK4/6 complexes all belong to the INK family of CDKIs (p15^INK4b^, p16^INK4a^, p18^INK4c^ and p19^INK4d^). Inhibitors of all other cyclin-CDK complexes belong to the Cip/Kip family of CDKIs (p21^Cip1^, p27^Kip1^, p57^Kip2^)

To understand the role of cell cycle proteins in AD pathology, it is crucial to understand how the cell cycle operates under healthy conditions (described in detail in [66]). The eukaryotic cell cycle can be divided into a gap 1 (G_1_) phase, DNA synthesis (S) phase, gap 2 (G_2_) phase and mitotic (M) phase (Fig. 1). If the environment is unfavourable of cell division, the cell can enter G_0_ phase, a state of prolonged cell cycle arrest. Cells can only enter G_0_ phase as long as they reside in G_1_ phase. Once the cell has passed G_1_ phase it is fully committed to the cell cycle and unable to return to G_0_ phase. To successfully proceed through the cell cycle, the cell needs to pass several checkpoints. Progression of the cell cycle past these checkpoints is closely monitored and regulated by a cell cycle control system [66]. Core components of this system are cyclin-dependent kinases (CDKs) that are activated upon binding to specific cyclin proteins. Active CDKs can phosphorylate downstream signalling proteins thereby stimulating progression through the different phases of the cell cycle. The activity of CDKs can be inhibited, on the other hand, by reduced transcription or enhanced degradation of cyclins, or by CDK inhibitor (CDKI) proteins from the Cip/Kip family or INK4 family. Each phase of the cell cycle is characterised by the involvement of specific CDKs, cyclins and CDKIs (Fig. 1).

It is believed that, during adulthood, neuronal cell division can only take place in the subventricular zone and subgranular zone of the human hippocampus [25]. Once neurons are fully differentiated, it is assumed they enter G_0_ phase, during which cell cycle re-entry is continuously blocked. It is therefore surprising that post-mortem studies report re-expression of cell cycle proteins in differentiated neurons of AD patients (Table 1). Whereas this re-expression has been shown to occur in different areas of the brain, the majority of studies have focused on post-mortem hippocampal tissue. Often, but not exclusively, cell cycle proteins are found to colocalize with NFTs [6, 7, 12, 15, 24, 39, 65, 91, 98, 105, 111, 114, 115] and senile plaques [6, 7, 12, 24, 39, 98, 111, 114, 115]. This observation suggests that aberrant expression of cell cycle proteins may be a pathological feature of AD. Furthermore, since re-expression of cell cycle proteins is already witnessed in patients with early AD pathology [71, 72] or mild cognitive impairment (MCI) [105], a prodromal stage of AD, it has been put forward that cell cycle abnormalities potentially play an early, or even causal role in AD pathogenesis. From post-mortem studies it seems that post-mitotic neurons can progress as far as G_2_/M phase; markers of G_1_ (CDK4, cyclin D, E and p38), DNA synthesis [69, 102] and S phase (cyclin A, PCNA, mcm2), G_2_ (CDK1) and M phase (cyclin B, CDK1) (Table 1) have been detected. However, it seems that differentiated neurons in AD patients cannot complete the cell cycle, as no studies report successful events of mitosis. It has therefore been hypothesised that post-mitotic neurons can proceed up until the G_2_/M phase, at which stage their progression is blocked and aborted. The observation that post-mitotic neurons express cell cycle proteins is not restricted to AD cases as several studies show re-expression of cell cycle proteins in other neurodegenerative disorders as well [71, 72, 89, 91].

**Table 1:** Overview of studies on cell cycle proteins in post-mortem human brain tissue

References	Areas	Reference tissue	AD patients (*N*)	HCs (*N*)	Increased in AD patients	No difference between AD patients and HCs	Colocalized with NFTs*	Colocalized with senile plaques*	HCs^†^
[56]	Temporal cortex	No	3	3	Cdc2 (CDK1)	MAP2	N/A	N/A	MAP2
[91]	Frontal cortex, medial temporal lobe, hippocampus (areas investigated were not the same for all subjects)	No	3	3	Ki-67		Yes	No	Ki-67
[71]	Hippocampus	No	AD = 5 Pre-AD = 7	3	Cyclin B1, cyclin E	Cyclin A, cyclin D1	No	No	Cyclin E
[72]	Hippocampus	No	AD = 4 Pre-AD = 9	4	Ki-67	PCNA	No	No	Ki-67
[65]	Hippocampus	No	8	4	CDK4		Yes	N/A	CDK4
[98]	Hippocampus	No	9	7	Cdc2 (CDK1), cyclin B1		Yes	Yes (cdc2)	cyclin B1
[15]	Hippocampus, brainstem, locus coeruleus, dorsal raphe nuclei, entorhinal cortex, inferotemporal cortex	Yes (Cerebellum)	12	8	Cyclin B1, cyclin D1, PCNA, CDK4		Yes (PCNA and cyclin B1)	N/A	PCNA, cyclin B1, CDK4
[89]	Hippocampus	No	57	3	Cyclin E	Cyclin A, cyclin B1, cyclin D1	N/A	N/A	cyclin E, cyclin B1
[114]	Hippocampus, frontal cortex	Yes (Cerebellum)	18	18	p38		Yes	Yes	P38
[115]	Hippocampus	No	22	47	CDK7 (age 54-65, > 78)	CDK7 (age 66-78)	Yes	Yes	CDK7
[24]	Hippocampus and temporal cortex	No	53	35	Cdc25a phosphatase (ICC)	Cdc25a phosphatase (IB)	Yes	Yes	Cdc25a phosphatase (IB)
[111]	Hippocampus, frontal cortex	Yes (Cerebellum)	11	7	p38		Yes	Yes	ERK
[37]	Temporal cortex	No	12	15		Cyclin D1, cyclin E (inverse correlation with Aβ deposits)	No	No	Cyclin D1, cyclin E
[39]	Temporal cortex	No	19	21	p38	pRb (significantly lower in AD Braak stage VI)	Yes (p38)	Yes (p38)	pRb
[105]	Hippocampus, nucleus basalis, entorhinal cortex	No	AD = 9, MCI = 10	6	Cyclin B1, cyclin D1, PCNA		Yes	N/A	Cyclin D1, PCNA
[12]	Hippocampus and “cortex”	No	10	8	mcm2		Yes	Yes	Mcm2
[20]	Cerebellar dentate nucleus and cerebellar cortex	No	22	19	Cyclin A, CDK4	PCNA	N/A	N/A	Cyclin A, CDK4, PCNA
Inhibitory regulators of the cell cycle									
[7]	Temporal cortex	No	6	6	p16^INK4a^		Yes	Yes	0
[65]	Hippocampus	No	8	4	p16^INK4a^		Yes	N/A	p16^INK4a^
[6]	Entire brain except brainstem and cerebellum	No	34	11	p15^INK4b^, p16^INK4a^, p18^INK4c^, p19^INK4d^	p21Cip1, p27Kip1	Yes (p15^INK4b^, p16^INK4a^, p18^INK4c^, p19^INK4d^)	Yes (p15^INK4b^, p16^INK4a^, p18^INK4c^, p19^INK4d^)	0

*AD* Alzheimer’s disease, *HCs* healthy controls, *IB* immunoblot, *ICC* immunocytochemistry, *N* number of subjects, *NFTs* neurofibrillary tangles, *N/A* not applicable, *0* no cell cycle markers stained in HCs

* Cell cycle markers were colocalized, but not exclusively, with NFTs/senile plaques

^†^This column indicates which cell cycle proteins were detected in healthy controls, even if expression levels were very low

The interpretation of the nature of the observed cell cycle proteins in neurons in post-mortem tissue remains complex. From post-mortem studies alone it is difficult to infer a functional relation between neuronal cell cycle re-entry and pathology. Post-mortem studies provide the researcher with a static picture obtained during the progression of pathology, which makes it difficult to draw conclusions about the functional sequence of events. Re-expression of cell cycle proteins by neurons could be pathology induced preceding neurodegeneration or an adaptive response to a changing cellular environment. In addition, studies on human brain tissue show that cell cycle proteins are expressed in healthy control cases as well [12, 15, 20, 24, 37, 39, 56, 65, 71, 72, 89, 91, 98, 105, 111, 114, 115] (Table 1), although generally at lower levels than in AD patients, and in neurons without apparent presence of pathology. These findings question the rarity, specificity and causality of cell cycle protein expression in AD. Moreover, the observed expression of cell cycle proteins in healthy adult neurons suggests that cell cycle proteins could fulfil essential physiological functions in post-mitotic neurons.

## Physiological functions of cell cycle proteins in post-mitotic neurons

### DNA Repair

Previous studies have indicated a role for cell cycle proteins in DNA repair. Oxidative stress comprises a major source for DNA damage in post-mitotic neurons. All aerobic organisms experience oxidative stress, which can occur as a side effect of the mitochondrial electron transport chain, (chronic) inflammation and ionizing radiation. It can be harmful to the cell due to the production of reactive oxygen species (ROS), chemically reactive molecules that contain oxygen. Excessive ROS can lead to double-strand breaks (DSBs) in DNA [44], which are considered to be the most lethal DNA lesions. Cells rely on two repair mechanisms when DSBs are detected: homologous recombination (HR) and non-homologous end joining (NHEJ) (reviewed in [62, 81]). The HR mechanism uses a homologous chromosome or an identical sister chromatid as a template to repair DSBs. Since sister chromatids are identical, and homologous chromosomes very similar to each other, this repair mechanism is usually without genetic risks. NHEJ, on the other hand, joins the ends of the broken DNA segments and, if necessary, fills the gaps. NHEJ is often considered imprecise, as deletions and insertions can more easily occur compared to when the HR repair mechanism is employed. It is believed that differentiated neurons primarily use NHEJ to repair DSBs [75].

Cell cycle control and DNA damage repair are intricately linked in cycling cells, so it might not be surprising that this is conserved to some extent in mature neurons. Cell cycle proteins have been shown to play a role in the activation and expression of components of DNA repair mechanisms (recently reviewed in [96] and [28]), providing a clue as to why post-mitotic neurons could reactivate their cell cycle machinery to carry out DNA repair processes. Evidence is now starting to suggest that cell cycle activation is an important feature of the NHEJ response in differentiated neurons [18, 83, 95]. After introducing DSBs in vitro by treatment with the ROS hydrogen peroxide, differentiated neurons showed increased expression of proteins related to the NHEJ response [95] and cell cycle entry, such as cyclin D1 [83], phosphorylated retinoblastoma protein (pRb) [83, 95], and more global cell cycle regulators such as Ki-67 [83, 95] and mcm2 [83]. Furthermore, increased phosphorylation of Rb by cyclin C and cyclin D was detected [95]. Preventing cyclin C-mediated cell cycle entry or simultaneous blocking of CDK4 and CDK6, both important during G_1_ progression (Fig. 1), augmented DNA damage upon hydrogen peroxide exposure [83, 95]. Interestingly, forced entry into G_1_ phase also activated the NHEJ response in the absence of DSB lesions [95]. Post-mitotic neurons subjected to repairable DNA damage, did not proceed to S phase, but remained in G_1_ phase [83]. On the other hand, insurmountable DNA damage induced by excess levels of hydrogen peroxide seemed to promote G_1_/S phase progression, as shown by increased bromodeoxyuridine (BrdU; a thymine analog) incorporation, CDK2 and cyclin E expression, and subsequently led to apoptosis [83]. Blocking CDK2 activity reduced apoptosis, but did not affect DNA repair [83]. These results were largely confirmed by an in vivo experiment, in which rats underwent sublethal ionizing radiation (IR), focused on their heads, to induce DSBs [18]. Sensory ganglion neurons of these animals were investigated at 0.5, 3, 6 h, 1, 3 and 15 days post-irradiation. DSBs were confirmed by immunostaining for phosphorylated H2AX and the presence of 53 binding protein 1, both involved in the NHEJ response [18]. A peak in nuclear cyclin D was found 1 day post-IR and, even though it decreased afterwards, it was found to remain elevated up to 15 days after radiation. Interestingly, p21, an inhibitor of G_1_/S phase progression also peaked 1 day post-IR, but decreased after 3 days and was absent at day 15. Finally, cyclin A expression could not be detected, supporting the notion that neurons did not progress to S phase. In line with this, neurons did not show signs of apoptosis. These findings support that sublethal DSBs cause differentiated neurons to re-enter G_1_ phase, but without subsequent progression to S phase.

More indirect support for a link between DSBs and cell cycle re-entry comes from studies on ataxia telangiectasia mutated (ATM). ATM autophosphorylates upon detection of DSBs [9] and is an important inhibitor of cell cycle phase transitions, including G_1_/S phase progression, to allow cycling cells time to repair DNA damage. The *Atm* gene is defective in patients with the disease ataxia telangiectasia (AT), which is characterised by decreased resistance to DSBs, progressive neurodegeneration of Purkinje cells and neuronal cell cycle protein expression [45, 103]. This illustrates that DNA damage-induced cell cycle arrest is not only important in cycling neurons to repair DNA, but might also be of great importance in post-mitotic neurons. An in vivo study using *Drosophila melanogaster* expressing human tau in the fly’s nervous system reported that tau-expressing post-mitotic neurons showed increased signs of DSBs compared to post-mitotic neurons in healthy flies [48]. Interestingly, decreasing ATM in tau-expressing neurons, increased apoptosis and PCNA expression [48]. This indicates that a subset of tau-expressing neurons, perhaps those with repairable DSBs, would naturally not enter S phase but might remain in G_1_ phase and therefore survive. Indeed, down-regulation of Cdh2 and p53, both involved in ATM-associated G_1_ arrest [19], also increased PCNA expression and apoptosis in tau-expressing neurons [48]. Hence, decreasing ATM activity and therefore cell cycle arrest at G_1_ phase might allow more neurons to progress into S phase and die. Some studies have suggested that *Atm* deletions can protect neurons from DNA damage-induced apoptosis, since ATM may be required for p53-mediated apoptosis [55]. Several other studies, however, also support that knockdown of ATM causes expression of S phase markers in post-mitotic neurons [58, 80, 103, 104] and enhance apoptosis [58, 80, 104], which both seem to increase under conditions of oxidative stress [104], confirming the neurodegenerative phenotype and susceptibility to cancer [68] seen in AT patients. Although these studies did not investigate if NHEJ repair occurred in these neurons during G_1_ phase, they do support a protective role for G_1_ arrest upon DNA damage in adult neurons.

In conclusion, it seems that post-mitotic neurons may be capable of re-entering the cell cycle to initiate DSB repair, and remain in G_1_ phase as long as DNA damage is repairable or sublethal. High doses of irreparable DSBs induce further progression to S phase in the cell cycle and ultimately lead to apoptosis. The link between S phase progression and apoptosis is supported by other studies [22, 48, 55]. The underlying mechanism responsible for apoptosis in S phase requires further study, but has been suggested to be due to replicative stress [108]. As cells will replicate their DNA during S phase, this will give rise to hyperploidy in neurons. Perhaps due to insufficient DSB repair, the majority of neurons presumably cannot complete S phase, which may cause the resulting aneuploidy to further enhance genomic instability and eventually causes the neuron to die.

### Neuroplasticity

Studies on cell cycle proteins have also suggested a link with neuroplasticity. Neuroplasticity refers to the ability of the brain to structurally and functionally adapt to its dynamic and continuously changing environment. Immunoelectron microscopy and immunoblotting experiments have shown an association of cyclin B and D, as well as CDK2 and CDK4 with the axonal microtubule cytoskeleton in mouse neocortical tissue [82]. The same study furthermore showed kinase activity to bovine tubulin, especially by cyclin B complexed with CDK2. siRNA-driven down-regulation and pharmacological inhibition of CDK1, 2 and 4, and cyclin B, D and E in vitro promoted neurite outgrowth in a mouse neuroblastoma cell line and mouse primary neurons, indicating a role for those cell cycle proteins in the regulation of network stability and neuronal cytoskeleton dynamics [82]. Accordingly, cyclin D1 was recently linked to microtubule reorganisation in hippocampal rat neurons [54]. Another recent study demonstrated that cyclin E could also play a role in the formation of synapses in post-mitotic neurons through inhibition of CDK5 [74]. Unlike other CDKs, who are generally activated by a cyclin protein, CDK5 is catalytically activated by p35/p39 or the more stable fragment p25 and exerts inhibitory control over the cell cycle [110]. Many studies have shown a key role for CDK5 in synaptic plasticity. However, as activation and inhibition of CDK5 have both been associated with improvement but also with impairment in measures of neuroplasticity, its functions are likely to be context dependent [21]. Cyclin E was demonstrated to inhibit CDK5 activity by preventing it from binding to p35/p39 [74]. Acute ablation of cyclin E in post-mitotic neurons led to a decreased number of synapses and dendritic spines in in vitro cultures of mouse hippocampal neurons [74]. These findings were confirmed in vivo, as cyclin E knockout mice showed alterations in dendritic spines, a reduced length of postsynaptic densities and decreased synaptic transmission. In addition, cyclin E null-brains showed less phosphorylation of the NR1 subunit of the NMDA receptor and, correspondingly, reduced NMDA-dependent currents.

As neurodegeneration leads to a loss of connections between neurons, this is an important stimulus to trigger neuroplastic processes in the remaining neurons. It could therefore well be that cell cycle proteins found to be upregulated in healthy neurons upon loss of synaptic connections aid in synaptic remodelling. Loss of synaptic connections has been shown to trigger expression of cell cycle proteins in intact post-mitotic neurons both in vitro [51] and in vivo [35, 36]. Lesions in the entorhinal cortex were found to induce cell cycle protein expression (cyclin D1, cyclin B and ERK1/2) in the dentate gyrus of the hippocampus [35], and vice versa (cyclin D1, CDK6, PCNA, CDK2, cyclin B, CDK5 and p25/35) [36]. CDK4 and its activator cyclin D1 were upregulated in intact neurons bordering ischemic cores in rat brain [60, 61] and were also found to be upregulated upon contusion in intact neurons without signs of apoptosis [46]. This all implies that these cell cycle proteins are necessary for survival, repair or compensatory mechanisms.

Several other cell cycle regulators have been linked to neuronal plasticity in fully differentiated neurons, such as the anaphase-promoting complex/cyclosome (APC/C) and its activator Cdh1, polo-like kinase 2 (Plk2), Aurora A kinase and the origin recognition complex (ORC) (reviewed in [29]). In the cell cycle, APC/C primarily functions to drive M phase progression and exit by controlling degradation of other cell cycle proteins, including cyclin B. In developing post-mitotic neurons, nuclear Cdh1-APC/C was demonstrated to suppress axonal growth [53], whereas cdc20-APC/C activity at the centrosome was shown to be critical for dendrite morphogenesis [49]. In addition, Cdh1-APC/C activity is suggested to play a role in maintaining homeostatic plasticity, the mechanism by which neurons adapt their spiking output within an optimal range following chronic excitation or depression, by down-regulating the Glur1 subunit of the AMPA receptor upon chronic elevated synaptic activity [31]. Another cell cycle protein involved in homeostatic plasticity is Plk2, which is active during S phase and late G_2_ phase. Plk2 was shown to bind and degrade CDK5-phosphorylated spine associated RapGAP protein (SPAR), which led to decreased synaptic strength [84]. Aurora A kinase, which mainly functions to coordinate centrosome dynamics, appears to play a role in neurite extension [67] and in NMDA activity dependent protein translation at the synapse [40]. Another major regulator of the cell cycle, ORC, is involved in dendritic branch and spine development [41]. Finally, it was shown that overexpression of constitutively active Ras, an important regulator of cell proliferation, led to altered synaptic connectivity at both the functional and structural level in cortical neurons of mice [5].

Altogether, above findings argue that cell cycle proteins may contribute to neuroplasticity. The exact mechanism underlying neuroplastic processes mediated by cell cycle regulators in post-mitotic neurons remains to be determined. As neuroplasticity can occur on many levels and has been shown to involve multiple cell cycle proteins, it is likely that various mechanisms can be employed in post-mitotic neurons depending on the contextual demands. The above studies suggest that cell cycle proteins might directly interact with the neuronal cytoskeleton, and that they may exert their effects by modulating specific functions of other regulators of cytoskeletal dynamics, such as CDK5 or SPAR. In addition, cell cycle proteins may affect glutamate receptor expression or protein synthesis. Alternatively, it has been hypothesised that neurons retract from the cell cycle to use their cell cycle machinery for neuroplastic purposes (reviewed in [3]). In line with this hypothesis, it has been suggested that the increase in cell cycle proteins reflects failed neuroplasticity in AD; neuroplastic signalling might be erroneously interpreted for mitogenic signalling and therefore activate the ancient mechanisms of the cell cycle in differentiated neurons leading to neurodegeneration [3].

## Cell cycle activation and neurodegeneration

Early indications that re-entry of the neuronal cell cycle could lead to neurodegeneration, came from a study over two decades ago [27]. In this study, the oncogene Tag was over expressed in post-mitotic, cerebellar Purkinje cells in mice. Rather than inducing tumorigenesis, the authors were confronted with increased neurodegeneration. Over the years, other experimental studies [57, 80], correlative evidence from post-mortem studies (Table 1), and the fact that cell cycle reactivation often accompanies Aβ-, and tau-mediated cell death have been supportive of this phenomenon [1, 22, 32, 43, 47].

In addition to DNA damage and replicative stress-induced apoptosis, several pathways have been proposed to explain the mechanism of cell cycle-mediated neurodegeneration. One hypothesis states that neurons die through ‘phase stasis’; in late S phase, G_2_ and M phase, mitochondrial proliferation takes place, exposing neurons with defective control over their cell cycle even more to the damaging effects of ROS [90]. Two other theories revolve around CDK1 as a mediator of cell death upon neuronal activity deprivation. Activity deprivation was demonstrated to induce E2F1-mediated CDK1 expression, which in turn was found to phosphorylate BAD at its serine 128 site [51, 52]. If not phosphorylated at serine sites 112 and 136, BAD heterodimerises with Bcl-2 and Bcl-X_L_, thereby promoting apoptosis [109]. Growth factors can induce phosphorylation of BAD at serine 112 and 136, which leads to interaction of BAD with proteins from the 14-3-3 family [109]. This in turn promotes neuronal survival. It has therefore been proposed that phosphorylation of BAD at serine 128 prevents sequestration of serine 136-phosphorylated BAD by 14-3-3 proteins and thereby antagonises growth factor-induced neuronal survival. More recently, the transcription factor FOXO1 was found to be phosphorylated by the cyclin B-CDK1 complex as well [107]. Phosphorylation resulted in the translocation of FOXO1 from the cytoplasm to the nucleus. In the nucleus, FOXO1 then induced expression of the pro-apoptotic gene *BIM.* Active CDK1 plays a role during G_2_ and M phase and both mechanisms therefore likely describe a different apoptotic pathway in post-mitotic neurons than is seen upon DNA damage-induced cell death.

It could be questioned, however, whether apoptosis is the primary underlying mechanism of cell cycle-related neurodegeneration in AD. Apoptosis is a relatively rapid way for neurons to die. AD on the other hand, is characterised by slow, but progressive neurodegeneration. Researchers have therefore been puzzled by the many neurons that were found to exhibit cell cycle markers in post-mortem tissue of MCI and AD patients. If these cell cycle proteins would indeed indicate that apoptotic processes are being at work, those neurons would be expected to die within a very short time frame and this would not match the rate of neurodegeneration in AD. Another source for researchers to feel hesitant towards the hypothesis of cell cycle-induced apoptosis is the lack of neurodegeneration in a number of mouse models despite an up-regulation of cell cycle proteins [59]. The ‘two-hit hypothesis’ provides an explanation for this and the long period between cell cycle abnormalities and cell death [112, 113]. It postulates that oxidative stress and cell cycle re-entry, with either one preceding the other, cooperate to induce cell death. According to this hypothesis, oxidative stress or mitogenic alterations can drive neurons into a new steady state, in which they still function normally but at the cost of permanent adaptive changes. These changes render neurons more vulnerable to a second insult. The expression of cell cycle markers therefore reflects a mitotic steady state, in which neurons are more vulnerable to ROS-induced damage, and does not necessarily indicate ongoing neurodegeneration. According to this hypothesis, however, oxidative stress and cell cycle abnormalities seem to be two independent processes. This is counteracted by the many studies that report cell cycle up-regulation upon both high and low levels of oxidative stress [11, 14, 18, 23, 50, 55, 83, 95]. In addition to DNA damage-induced cell cycle re-entry described above, ROS have been linked to cell cycle activation in neurons via p38 activation, the induction of growth factors, and the inhibition of histone deacetylation (summarised in [50]). Finally, several dietary compounds affecting susceptibility to oxidative stress have also been shown to regulate cell cycle activation, such as selenium [78], iron [22, 26], and folic acid/homocysteine [55, 73]. Overall, these results seem to support a relationship between oxidative stress and cell cycle activation.

## Cell cycle proteins: significance in AD pathology

**Fig. 2:**
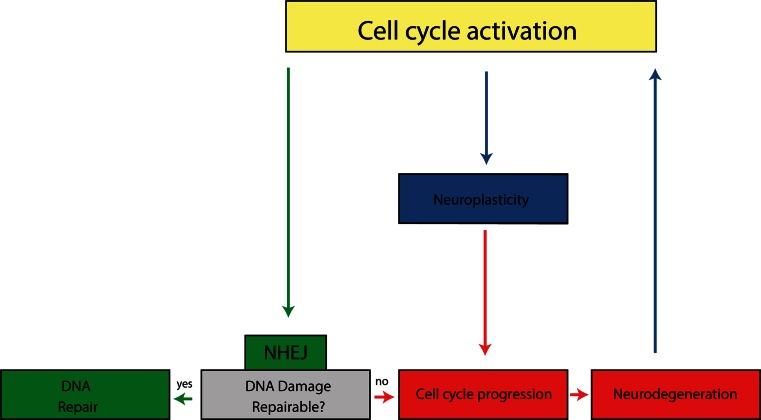
Cell cycle activation in post-mitotic neurons of AD patients. G_1_-phase entry of the cell cycle in post-mitotic neurons facilitates DNA repair via the non-homologous end joining (NHEJ) pathway (*green arrows*). DNA damage beyond repair will drive neurons to progress to S phase in the cell cycle which will ultimately result in neurodegeneration (*red arrows*). Accumulating AD pathology and neurodegeneration will increase the need for cell cycle-related neuroplasticity (*blue arrows*). Finally, neuroplastic signals may be misinterpreted for mitogenic signalling in AD patients, which also leads to neurodegeneration

It seems evident that cell cycle proteins can fulfil physiological functions in post-mitotic neurons, which include DSB repair and neuroplasticity. Both processes are very important throughout the different stages of AD to counteract pathology. We therefore propose that during the earliest, preclinical stages of AD, cell cycle protein expression may predominantly reflect repair of sublethal DSBs. However, with the accumulation of DSBs and AD pathology during disease progression, cell cycle-mediated neuroplasticity and neurodegeneration may become more predominant. Furthermore, control over DSB repair and neuroplasticity may become increasingly defective and this may also contribute to neurodegeneration (Fig. 2).

Firstly, we propose that the earliest stages of AD might be characterised by cell cycle protein expression to aid DNA repair. Preclinical and prodromal stages of AD are presumably featured by accumulating, yet repairable or sublethal DSBs. These DSBs might be caused by gradually increasing genotoxic insults and deficient DNA repair mechanisms. Indeed, oxidative DNA damage increases with age, but appears to be even further augmented in MCI patients [99]. Another potent candidate in AD that could partly be responsible for the occurrence of DSBs is Aβ [94]. Preclinical stages of AD are likely marked by elevations in oligomeric, nonfibrillar Aβ, which may contribute to the accumulation of DSBs [94] and therefore evoke re-expression of cell cycle proteins to initiate DNA repair. In line with this, in transgenic mice expressing human amyloid precursor protein with the Swedish (K670M/N671L) mutation, cell cycle changes were observed well before amyloid plaque formation [97, 106]. It was furthermore shown that Aβ oligomers could induce neuronal cell cycle entry in vitro as measured by cyclin D1 [86], BrdU incorporation [10, 97] and PCNA expression [10]. Interestingly, BrdU incorporation and PCNA expression increased in a concentration-dependent fashion [10, 97]. Similarly, it was demonstrated in vitro that fibrillar Aβ can induce hippocampal adult neurons to re-enter the cell cycle towards different stages, depending on the concentration [63]; whereas low concentrations of Aβ induced cyclin D1 expression, higher concentrations led to cyclin B1 expression.

Aβ has also been found to interfere with NHEJ [17]. This is supported by studies that have reported reduced NHEJ efficiency in AD patients [87], as measured by end-joining activity and the expression of Ku and DNA-dependent protein kinases—proteins responsible for recognition and binding to DSBs to facilitate bridging of the DNA ends. Cell cycle reactivation might, however, still occur to initiate the NHEJ response, which will later on lack effectiveness. It could then be hypothesised that cell cycle activity is further increased in an attempt to compensate for an inefficient repair mechanism. Altogether, this could account for results found in post-mortem studies where G_1_ phase markers are elevated in neurons in early stages of AD pathology [37, 38, 71]. To the best of our knowledge, no studies have addressed the spatial relationship between DSB lesions and cell cycle markers in post-mortem human brain tissue. One study investigated whether a correlation exists between markers of DSB repair and expression of cyclin A in post-mitotic cerebellar and hippocampal neurons of AD patients [20]. Even though the presence of DSB repair markers was positively correlated with diagnosis (AD vs. control), no correlation between cyclin A and DSB repair could be confirmed. The lack of correlation might be explained by the fact that DSB repair primarily takes place during G_1_ phase. It would therefore be interesting for future studies to investigate colocalization of NHEJ markers with G_1_ phase markers, or markers of DSB lesions with cell cycle stages.

Several experimental studies have shown that Aβ and tau can be mitogenic [1, 10, 22, 30, 32, 43, 47, 63, 86, 97], and that Aβ and tau-induced cell death is often mediated by cell cycle activation [1, 22, 32, 43, 47]. This suggests a functional relation between the accumulation of Aβ and tau during disease progression and the occurrence of cell cycle proteins in neurons. However, in human pathology the relation between cell cycle proteins in neurons and the pathological hallmarks of AD is not clear. Even though cell cycle proteins are often found to colocalize with NFTs, they are also found in neurons without the presence of neurofibrillary pathology. The severity of AD pathology can be indicated by Braak staging, which follows the progression of neurofibrillary changes in AD brain [13]. Whereas in early Braak stages the temporal cortex is almost devoid of neurofibrillary changes, increased presence of G_1_ phase markers is observed in this area of the brain [37, 38]. It also seems that the correlation between the presence of amyloid plaques and the occurrence of cell cycle proteins is not consistent (Table 1). Post-mortem studies have, however, not addressed the relationship between soluble, nonfibrillar Aβ and cell cycle activation. Since oligomeric Aβ has been linked to cell cycle protein expression and the induction of DSBs, this will be an interesting topic for further research.

According to Braak staging the emergence of neuronal cell cycle protein expression is prominent in stages that precede the occurrence of plaques and tangles. This supports our hypothesis that re-expression of cell cycle proteins is an early event in AD, and could be associated with an adaptive response related to DNA repair or neuroplasticity. We next put forward that with disease progression, the building up of oxidative stress, genotoxic insults and deterioration of DNA repair mechanisms lead to the accumulation of DSB lesions, which have been found to be increased in AD patients [20, 88], eventually to an extent that is no longer repairable. This will increasingly drive neurons to progress to more advanced stages in the cell cycle, as will be reflected by increased expression of S phase markers, and eventually result in neurodegeneration (Fig. 2). A rise in cyclin E, involved in G_1_/S phase transition, has indeed been reported in the dentate gyrus, subiculum, CA2 and CA4 of the hippocampus of AD patients compared to pre-AD patients [71]. Another study reported a similar trend for Ki-67 in the CA2, CA3 and CA4 of the hippocampus, whereas an inverse relationship with disease stage was seen in the subiculum, dentate gyrus and CA1 of the hippocampus [91]. Finally, in transgenic mice carrying the Swedish (K670M/N671L) mutation of the human amyloid precursor protein, a dramatic increase in cyclin D1 and cyclin A was witnessed between 6 months and 12 months of age in frontal cortical layers V/VI [97].

Lastly, we propose that with the development of AD pathology and loss of synaptic connections, the demand for neuroplasticity is gradually increased, as will also be reflected in cell cycle protein expression (Fig. 2). In line with this, cell cycle proteins were found to be upregulated in hippocampal areas of preclinical AD patients that did not match the expected pattern of neurodegeneration [72]. Additionally, cyclin E expression in the dentate gyrus was found to be significantly correlated with neuritic plaque load in the neocortex [71]. Neurodegeneration in one area eventually also results in activity deprivation-induced cell death in connected areas, which will be reflected in increased cyclin B-CDK1 complex expression [51, 52, 107]. In keeping with this, cyclin B was found to be elevated in the dentate gyrus of full-blown AD patients compared to pre-AD patients [71]. Finally, as neuroplastic signals might be (increasingly) misinterpreted for mitogenic signalling in AD post-mitotic neurons, cell cycle activation and neurodegeneration are even further enhanced [3].

An important question is whether cell cycle proteins in neurons can carry out their functions independent of full cell cycle re-entry. Using fluorescent in situ hybridization (FISH) at least partial chromosomal replication has been shown to be increased in neurons in AD brain tissue [69, 102]. In addition, increased numbers of neurons with a more-than-diploid content of DNA are most notable in prodromal and mild stages of AD [4]. Fully or partial replication of separate genetic loci on different chromosomes has been observed in hippocampal and basal forebrain neurons in AD cases while these abnormalities were absent in age-matched controls [102]. However, aneuploidy also occurs in healthy human brain tissue [42, 69, 79]. These signs of aneuploidy could indicate that post-mitotic neurons can truly re-enter the cell cycle, although sometimes leading to incomplete DNA replication. However, aneuploidy in post-mitotic neurons could also arise due to failed DNA replication in a neuronal progenitor cell. To differentiate between these two causes, the link between aneuploidy and cyclin B1 has been investigated, as the combination of the two would indicate an active cycle [69]. The majority of neurons in AD with a tetraploid content of DNA express cyclin B1, whereas this seemed not to be the case for healthy controls. The association between an elevated content of DNA and expression of cyclin B1 in AD indicates that some neurons have reactivated their cell cycle and progressed toward S phase and beyond [69]. This has been challenged by another observation that tetraploid nuclei are similarly prevalent in AD and control brains and are exclusively non-neuronal [100], thus suggesting that differentiated neurons could not fully replicate their DNA during S phase. However, cell cycle markers of G_2_ and early M phase have been repetitively observed in post-mitotic neurons, suggesting that these neurons have completed S phase. An explanation for this paradox might be that neurons could reach G_2_/M phase without completely replicating their DNA [108]. Forthcoming studies are therefore advised to further investigate the mechanism of cell cycle protein expression in relation to the physiological functions they fulfil in post-mitotic neurons, by addressing the occurrence of aneuploid neurons in the brain using a combination of appropriate markers and specifically address the different stages of AD pathology.

## Concluding remarks

Here we propose that cell cycle proteins may play a key role in DSB repair and neuroplasticity and that aberrant control of these physiological functions may eventually contribute to cell cycle-mediated neurodegeneration in AD (Fig. 2). The observed neuronal expression of cell cycle proteins in early pathological and preclinical stages of AD could represent an increased need for DSB repair. With disease progression, DSBs will gradually accumulate in post-mitotic neurons to a point where they can no longer be repaired. Consequently, these neurons will progress to more advanced stages in the cell cycle and eventually degenerate. The accumulating pathology and progressive neurodegeneration increase the need for neuroplasticity, as will also be reflected in the expression of cell cycle proteins in fairly intact neurons. In addition, aberrant activation of signalling pathways involved in neuroplasticity in AD neurons might also contribute to cell cycle-mediated neurodegeneration. This broad involvement of cell cycle proteins could explain the incongruence between the large amount of neurons expressing cell cycle markers and the estimated rate of neurodegeneration in AD [102, 105], as not all cell cycle proteins should be interpreted as predictors of neuronal cell loss. Since the balance between DNA repair, neuroplasticity and neurodegeneration may change depending on disease stage, it can be argued that each AD stage could be characterised by a specific pattern of cell cycle protein expression in post-mitotic neurons.

Considering a physiological role for cell cycle proteins in the early stages of AD, several aspects require attention in future studies. First, little is known about NHEJ activity and DSB prevalence in the different stages of AD. In addition, the spatial overlap between DSBs and cell cycle markers has unfortunately been poorly addressed in previous post-mortem studies. Future studies will therefore need to address the relationship between cell cycle markers and DSBs in post-mortem brain tissue, with specific attention to the NHEJ response and DSB prevalence in the different stages of AD pathology. Second, cell cycle proteins characteristic of G_2_ phase and M phase have been reported in control cases [12, 15, 20, 65, 98], early AD cases [72, 105] and neurons without apparent pathology [15, 20, 71]. Because their expression is unlikely attributable to DNA repair, prospective studies need to elucidate whether G_2_ and M phase markers can be ascribed to neuroplastic events or (age-related) neurodegeneration. Subcellular distribution of cell cycle proteins and colocalization with markers for neurodegeneration and neuroplasticity might provide clues as to which mechanism is being employed. And third, future studies are advised to investigate whether different types of aneuploidy, such as triploidy and chromosome 21 trisomy, can be correlated with protein expression of specific cell cycle stages and markers of apoptosis, DSBs and neuroplasticity. This will ultimately tell us if full cell cycle activation is required to initiate or carry out neurodegenerative processes or neuroplastic events.

A potential approach to obtain more insight in the role of cell cycle control and related physiological mechanisms during disease progression is proteomics on disease staged human brain samples. Previous proteomic studies on human brain tissue have shown the potential of detecting proteins associated with the cell cycle, oxidative stress and cell death [2, 64, 70]. Proteomics on human brain tissue samples could provide increased insight in the correlation of neurodegeneration and deregulation of the cell cycle in AD in different stages of the disease. A common problem in analysing these data with bioinformatics tools is that it is hard to estimate the relative contribution of the different cell types present in the original sample. Especially in investigating neuronal cell cycle changes, one wants to rule out cell cycle changes occurring in glia or other cell types than neurons. Bioinformatics tools are available to estimate the relative role of cell population using databases of transcripts enriched in astrocytes, neurons, and oligodendrocytes [16, 33]. Whether this could work for generic pathways like the cell cycle remains questionable. A more promising approach would be proteomic analysis of isolated neurons using laser capture microscopy (LCM) or fluorescence activated cell sorting (FACS). Current technical advances in this field have overcome problems in obtaining enough material using these approaches and the yield of identified proteins with proteomics. A clear advantage of this approach is that neurons labelled for specific cell cycle markers can be isolated and analysed.

In conclusion, increased cell cycle protein expression occurs in post-mitotic neurons during the progression of AD. Whereas the role of cell cycle proteins in neurons in disease mechanisms associated with apoptosis is widely recognised, their physiological functions are underappreciated. Here we discussed two physiological functions for cell cycle proteins in post-mitotic neurons, i.e. DNA repair and neuroplasticity. Aberrant control of these processes may, in turn, trigger cell cycle-mediated neurodegeneration. Understanding the physiological and pathophysiological role of cell cycle proteins in AD could give us more insight in the neurodegenerative process in AD.
